# Affordable Embroidered EMG Electrodes for Myoelectric Control of Prostheses: A Pilot Study

**DOI:** 10.3390/s21155245

**Published:** 2021-08-03

**Authors:** Ernest N. Kamavuako, Mitchell Brown, Xinqi Bao, Ines Chihi, Samuel Pitou, Matthew Howard

**Affiliations:** 1 Department of Engineering, King’s College London, London WC2R 2LS, UK; mitchell.brown@kcl.ac.uk (M.B.); xinqi.bao@kcl.ac.uk (X.B.); samuel.pitou@kcl.ac.uk (S.P.); matthew.j.howard@kcl.ac.uk (M.H.); 2 Faculté de Médecine, Université de Kindu, Kindu, DR, Congo; 3 National Engineering School of Bizerta, Carthage University, Tunis 2070, Tunisia; ines.chihi@uni.lu; 4 Department of Engineering (DOE), The Faculty of Science, Technology and Medicine (FSTM), University of Luxembourg, 4365 Luxembourg, Luxembourg

**Keywords:** myoelectric prostheses, embroidered EMG electrodes, pilot study, online and offline performance, conventional gel electrodes

## Abstract

Commercial myoelectric prostheses are costly to purchase and maintain, making their provision challenging for developing countries. Recent research indicates that embroidered EMG electrodes may provide a more affordable alternative to the sensors used in current prostheses. This pilot study investigates the usability of such electrodes for myoelectric control by comparing online and offline performance against conventional gel electrodes. Offline performance is evaluated through the classification of nine different hand and wrist gestures. Online performance is assessed with a crossover two-degree-of-freedom real-time experiment using Fitts’ Law. Two performance metrics (Throughput and Completion Rate) are used to quantify usability. The mean classification accuracy of the nine gestures is approximately 98% for subject-specific models trained on both gel and embroidered electrode offline data from individual subjects, and 97% and 96% for general models trained on gel and embroidered offline data, respectively, from all subjects. Throughput (0.3 bits/s) and completion rate (95–97%) are similar in the online test. Results indicate that embroidered electrodes can achieve similar performance to gel electrodes paving the way for low-cost myoelectric prostheses.

## 1. Introduction

Upper extremity loss is a highly disabling family of injuries that ranges from partial hand loss to loss of an entire arm. It can dramatically reduce a person’s quality of life by impairing their ability to interact with their environment creating an economic and social burden. The total number of people with upper-limb loss is often difficult to quantify because many countries do not keep track of the incidence of amputation. However, it is estimated that over half a million people were living with some degree of upper limb loss in the United States in 2005. This figure is projected to double by 2050 [1]. Upper limb loss is estimated to be even more prevalent in the developing world, with most of the world’s amputees and disabled living in low- and middle-income countries [2]. 

Prostheses, or artificial limbs, can replace lost functionality and improve the quality of life in people who have suffered upper limb loss. Historically, artificial upper limbs have been either cosmetic devices that restore the natural appearance of the lost limb or body-powered prosthetics that offer a limited restoration of functionality. Over the last century, however, various active artificial upper limbs have entered the literature and, increasingly, the commercial market. Most commercial functional prostheses, such as the Michelangelo hand (Otto Bock, Duderstadt, Germany) and the LUKE arm (Mobius Bionics, New Hampshire USA), are expensive devices for wealthy individuals in developed countries. However, a growing number of projects across the world aimed at harnessing the emerging technologies of the 4th industrial revolution, such as 3D printing, to produce affordable active prostheses. The Hero Arm (Open Bionics, Bristol, UK) and the Touch hand (Touch Prosthetics, Cape Town, South Africa) are examples of affordable active prostheses aimed at high-income and low- to middle-income nations, respectively. 

An active prosthetic may be further classified as either an electric or myoelectric device. The former is controlled using external buttons and joysticks, while the latter is controlled using electromyographic (EMG) signals [3]. In myoelectric control, time and frequency domain features are extracted from the user via surface electrodes and are then fed into a control algorithm. The algorithm, in turn, converts them into motor commands fed into the prosthesis, thereby allowing the user to control the prosthetic and replace some of their lost functionality [4]. 

The development of a more affordable surface EMG electrode is one way that the cost of producing and maintaining myoelectric-controlled artificial limbs can be reduced and is an area that has received less attention than other prosthetic components. The two most common electrode types used in surface EMG are solid metal dry electrodes, which are widely used in prosthetics [5], and Ag-AgCl gel’ wet’ electrodes, which are the standard in clinical measurements [6]. 

Gel electrodes have lower impedance than other surface electrodes due to the large contact area provided by the electrolyte gel, resulting in minimal impedance and noise. The adhesive coating on the electrodes also produces a stable skin–electrode contact, which minimises motion artefacts [6]. Despite their high signal quality, gel electrodes are ill-suited for the heavy use required in powered prosthetics. The electrolyte gel dries out over time, causing the signal to decay and eventually fail, after which the electrodes or gel must be disposed of and replaced. Furthermore, the adhesive coating used in the electrodes can irritate the skin during use. 

The solid metal dry electrodes used in current prostheses are typically made from stainless steel or titanium with a flat or domed shape. They are usually active with a preamplifier and other circuitry built directly into the electrode. These electrodes solve some of the problems posed by gel electrodes: they can be used for prolonged periods without signal failure, are reusable, and do not require adhesive coatings. However, they have shortcomings of their own. The metal plates can cause skin irritation through friction, and they are more susceptible to motion artefacts than gel-based designs. They are also more expensive to manufacture than gel and other electrode designs.

E-textiles may provide an alternative to the commercial electrode designs currently on the market. Textile electrodes can be constructed by embedding conductive fibres into a textile substrate using traditional fabrication techniques, such as embroidery and weaving [7]. Several textile electrodes for EMG have been presented in the literature [8,9,10], and their potential performance in myoelectric control has been demonstrated in wrist and hand gesture EMG classification experiments [11,12]. Because they are soft and breathable and do not use any abrasive chemicals, these textile electrodes do not irritate the user’s skin. Their flexibility enables the electrode to conform to the shape of the arm and ensures that solid skin contact is maintained throughout the measurement, thereby minimising motion artefacts. Embroidered electrodes can be easily integrated into commercial prosthesis sockets [13] or fixed locations on smart garments or sleeves, making the process of placing the electrodes simple and appropriate for use by amputees. Like other dry electrodes, the textile designs are also reusable and can be worn for long periods without signal failure. The use of silver-coated thread has also been shown to have an antibacterial effect that helps to keep electrodes hygienic [14]. 

This paper evaluates the usability of affordable embroidered textile electrodes in real-time myoelectric control against a gel electrode standard. The significant advantage of this textile electrode design is its simple construction, which could potentially enable it to be handmade [15]. The ability to produce the electrodes by hand, coupled with the advantages inherent to textile electrodes, such as reusability, could make the design as much as 40% cheaper than gel alternatives [16] and ultimately facilitate the construction of more affordable myoelectric prostheses. 

## 2. Materials and Methods

### 2.1. Embroidered Electrode Design

**Figure 1:**
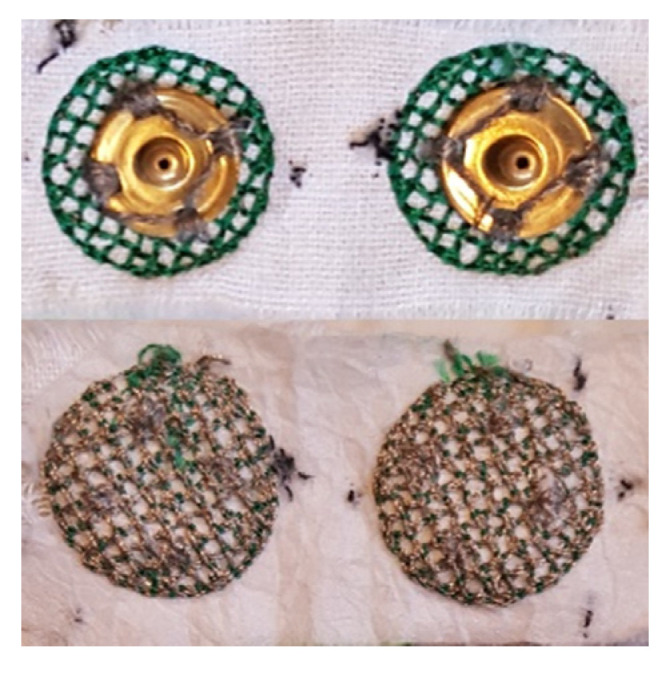
Example of embroidered electrodes used in this study. The outer side shows snap connectors (**top**), and the inner side shows conductive thread (**bottom**).

The electrodes were developed in the Centre for Robotics Research (CORE) at King’s College London in a series of investigations [15,16,17,18,19] and consist of silver-coated thread (Electro-Fashion Conductive Thread, Kitronik, 40 Ωm^−1^) embroidered into linen fabric with a Vilene cut away stabiliser. The embroidered pattern used is a 20 mm diameter circle with a cross-hatched fill pattern. The hatching has a 2 mm separation, and two iterations of embroidery are performed. An example of an electrode pair can be seen in Figure 1. The design parameters are selected to minimise resistance through the electrode in accordance with [18]. The electrodes are implemented in bipolar pairs with an inter-electrode distance (IED) of 25 mm. A standard snap fastener (Hemline, 13 mm, brass rust-proof fastener) is sewn onto each electrode and used to connect the electrode to an external amplifier. The electrodes are manufactured using a Pfaff Creative 3.0 (Pfaff, Kaiserslautern Germany) programmable sewing machine designed in the companion 6D Embroidery Software application.

### 2.2. Subjects

Three subjects volunteered for the experiments reported here, two female and one male. The participants were healthy, normally limbed, and ranged in age from 21 to 24. Due to the pandemic, we are not allowed to recruit more subjects, and thus this is a pilot study. We are planning to run a follow-up study soon with proper statistical analysis. All provided written informed consent before testing, and all tests had King’s College Research Ethics Committee approval (Approval number: LRS-16/17-4213).

### 2.3. Experimental Setup

**Figure 2:**
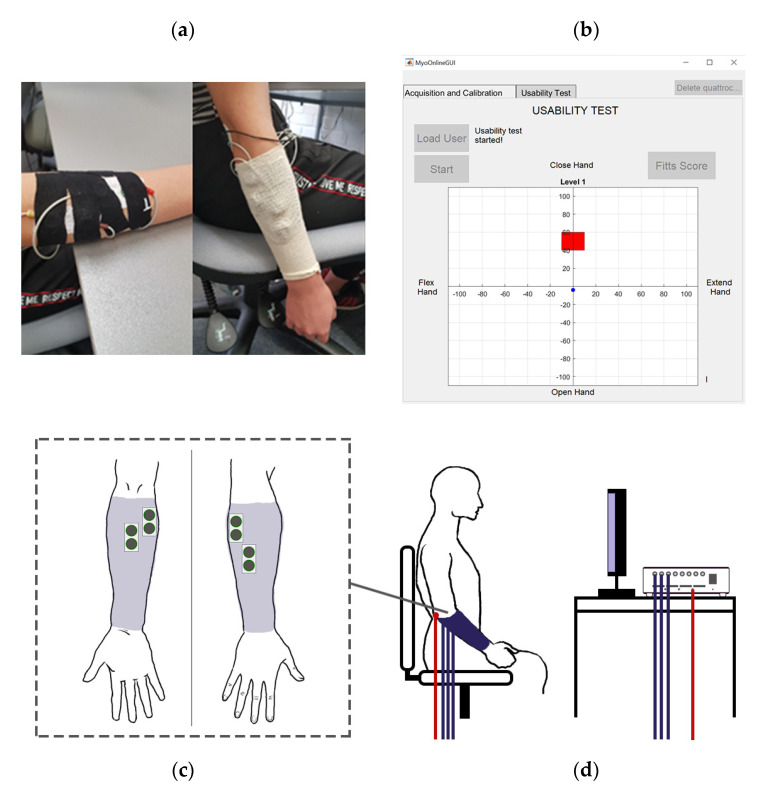
Experimental setup. (**a**) The electrodes are first placed with kinesiology tape and then secured with a bandage. (**b**) Usability test with the blue cursor and red target. (**c**) Positions of electrodes on the anterior and posterior sides of the right forearm. (**d**) Subject arrangement.

Two sets of surface electrodes are used for the experiments, one set of gel electrodes and one set of embroidered textile electrodes. When applying each set, subjects are seated, and four bipolar electrode pairs are placed on the forearm. Two pairs are placed over the extensor muscle group—the extensor carpi ulnaris and extensor digitorum—and two over the flexor group—the flexor carpi ulnaris and flexor carpi radialis. For experimental convenience, the textile electrodes are secured using kinesiology tape and covered using a tubular bandage. Alcohol wipes are used to clean the target areas before the application of the electrodes to ensure a clean connection following SENIAM recommendations [6]. For both sets, an additional reference gel electrode is attached at the elbow. The arrangement can be seen in Figure 2.

Each electrode is connected via snap connectors to a Quattracento amplifier (OT Bioelettronica, Torino, Italy), and the EMG signals are amplified with a gain of 1500, band-pass filtered (bandwidth 10–500 Hz), sampled at 2048 Hz, and A/D converted on 16 bits.

### 2.4. Experimental Protocol

The protocol consists of an offline test and an online usability test, both of which are conducted using a custom application implemented in Matlab 2019b (Mathworks, Natick, MA, USA).

**Figure 3:**
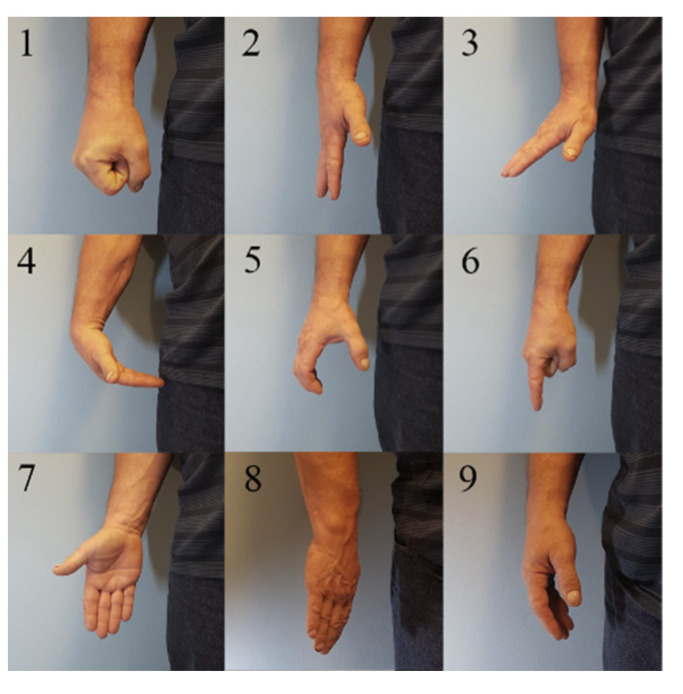
Gestures used in offline experiments. (1) closed hand, (2) open hand, (3) wrist extension, (4) wrist flexion, (5) chuck grip, (6) index point, (7) supination (8) pronation, and (9) rest.

For the offline test, the patients are taken through a series of nine gestures: closed hand, open hand, wrist extension, wrist flexion, chuck grip, index pointing, supination, pronation, and rest. These can be seen in Figure 3. Each gesture is performed four times, each time for six seconds following a three-second count down, which allows the subject to prepare. There is a six-second rest period between each gesture to prevent muscle fatigue.

For the usability test, only the first four gestures, and rest, are used. The subjects are again asked to perform each motion for six seconds following a three-second countdown with six seconds’ rest between gestures, but now each gesture is performed three times. The recorded measurements are used to train an artificial neural network (ANN) for gestures classification (see Section 2.5). The subjects are then asked to complete a game in which they move a cursor to a series of square target areas on a two-dimensional grid (see Figure 2b). Both grid axes range from −110 to +110 units, and each combination of axis and direction is associated with one of four gestures, while rest causes the cursor to remain in its previous position on the grid. For each target, the cursor begins at the origin. The subject can move the cursor a fixed step size in one of the four orthogonal directions by performing the associated gesture. The subject was asked to hold the position within the target for one second (dwell time) for the trial to be considered successful. If the subject was unable to reach the target within a 20 s time limit, the online trial was considered unsuccessful, and the participant moved on to the next target with the cursor back at the origin.

There are 24 targets in a complete game, and six configurations for the width and distance from the origin of the targets, each of which is used four times during a game and has a particular index of difficulty. Each configuration is associated with four target numbers between 1 and 24, and, before the start of the game, MatLab’s randperm() function is used to randomise an integer array from 1 to 24 using pseudo uniform random number generation, which then determines the order in which targets will appear. Table 1 shows the width and distance combinations for each configuration.

The step size for each gesture classification is 110/28. The numerator is the total length of the axis in each direction, so each step is a fixed fraction of the length. At the same time, the denominator was chosen through trial and error. Subjects complete the game three times.

The order of the electrode type (gel first or textile first) was controlled through counterbalancing. The first subject was randomly assigned to one order using a coin flip, and the order was reversed with each subsequent subject. In practice, because there are only three subjects, two subjects started in one condition, and one in the other.

### 2.5. Signal Processing

All offline data are processed using the same procedure. The first and last second of each EMG recording in a dataset is removed to account for subjects beginning and ending each gesture, leaving a four-second sample of each gesture. The sample for each gesture is pre-processed by subtracting the mean value in each channel was s. The sample is then segmented with a 200 ms window, which is passed over the sample such that each segment has a 50 ms overlap with the preceding segment. Six features are extracted from each segment: zero crossings, root mean square, mean absolute value, waveform length, slope sign change, and Willison amplitude. The feature matrix dimensionality reduction using principal component analysis (PCA) with a 0.95 variance threshold typically reduced the number of features to four.

The offline classification performance is evaluated using 10-fold cross-validation. For each subject, the raw EMG data for each electrode type is pooled, and pre-processing, feature extraction, and dimensionality reduction is performed. The prepared data are then divided into training (80%), validation (10%), and testing sets (10%), with the test set being the k^th^ fold of the cross-validation. Individual subject models are trained using a feedforward ANN with a single 18-node hidden layer with tansig transfer function and nine-node softmax output layer. Levenberg–Marquardt error backpropagation is used for training with validation-based early stopping for regularisation. This is repeated for each fold, and the mean and standard deviation of the test fold accuracies is taken. The individual subject data is then pooled together into all gel and all fabric datasets, and the above cross-validation procedure is repeated to evaluate a general model for each electrode type.

For the usability test, a similar processing procedure is used as in the offline test to train an ANN for each subject. Online pattern recognition is achieved by recording 200 ms samples and feeding each sample to the trained algorithm for real-time classification. A Fitts’ Law approach is used to assess real-time performance in each round of the online game, and the completion rate and throughput are calculated as performance metrics. The completion rate is defined as the percentage of targets successfully reached out of the total number of targets. Throughput (TP) is the amount of information transmitted by the user via the EMG electrodes. This can be expressed mathematically as the average ratio of the index of difficulty (*I*) of each target and the completion time (*C*), which is the time required to reach each target:

(1) $$T=\frac{I}{C}.$$

The *I* for the usability test is defined according to the distance of the target centres from the origin (*D*) and the width of the target area (*W*) and is derived from Shannon’s extension of Fitts’ law in accordance with [20]:

(2) $$I=log_{2}\;\left(\frac{D}{W}+1\right)$$

The values of *I* for the targets used in the experiment can be found in Table 1.

## 3. Results

**Figure 4:**
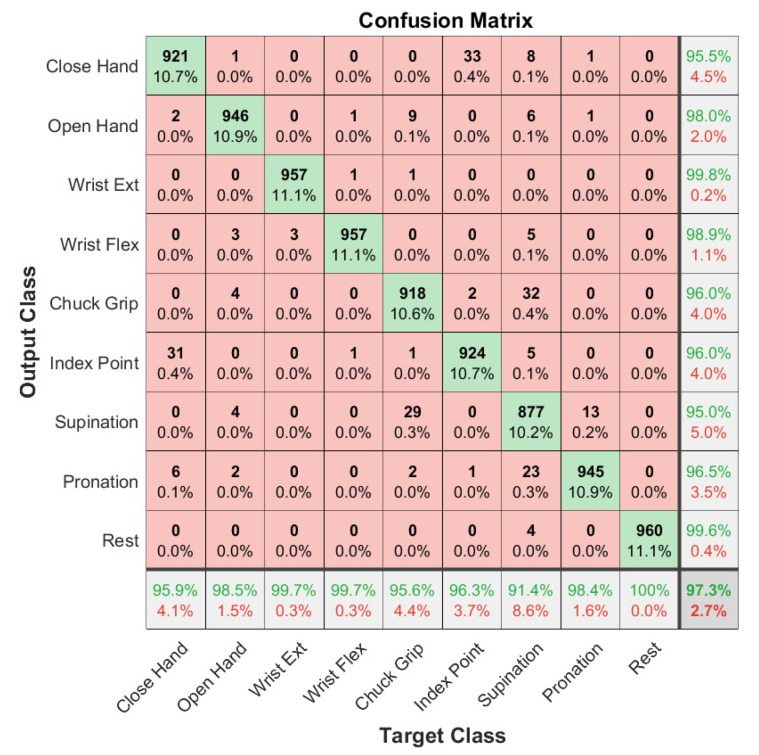
Confusion matrix for 10-fold cross-validation of a model trained on combined gel data from all subjects. The bottom row is the recall, the right column is the precision, and the bottom right is the overall accuracy.

**Figure 5:**
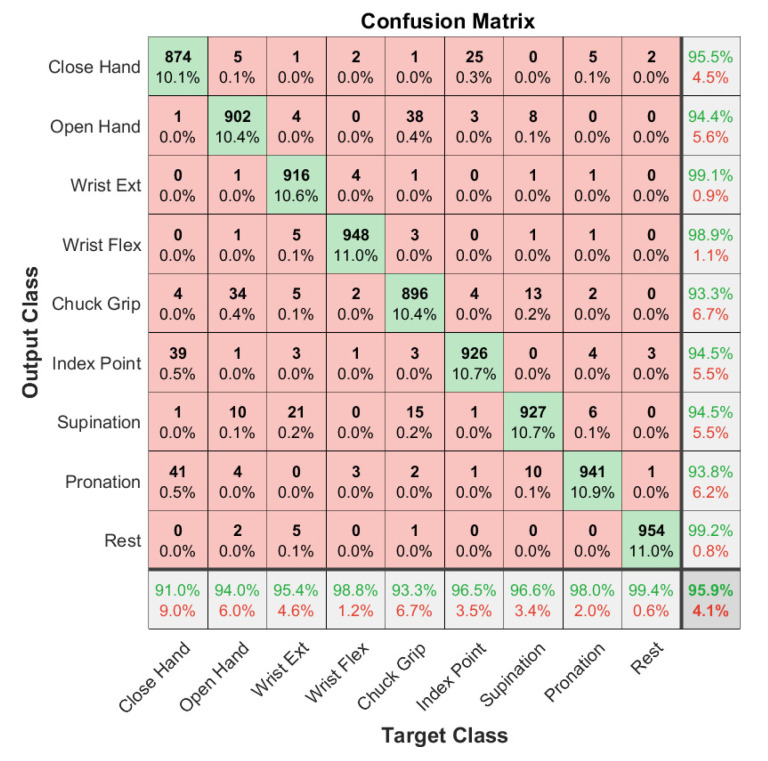
Confusion matrix for 10-fold cross-validation of a model trained on combined fabric data from three subjects. The bottom row is the recall, the right column is the precision, and the bottom right is the overall accuracy.

Offline models were trained and evaluated using the individual subject datasets for each electrode type and on a pooled dataset containing all electrode types. Table 2 shows the offline classification accuracy of models trained on individual subject data and their means and standard deviations. The mean accuracy across the three subject-specific models is 98.4% for the gel electrodes and 97.9% for the embroidered electrodes overall nine gestures. The classification accuracy for the general model trained on all gel electrode data is 97.3 ± 1.2%, and the accuracy for the model trained on all embroidered electrode data is 95.9 ± 1.2%. These can be seen in the respective confusion matrices (Figure 4 and Figure 5). The percentages in the main (red and green) square are total classifications made across all classes, so they sum to 100. Therefore, the numbers on the diagonal are the number of correct classifications for each class as a percentage of total classifications made. There are nine classes, so they would each be ~11.1% if the model classified each class perfectly. The accuracy in practice is close to this for both models, reflecting good accuracy.

**Figure 6:**
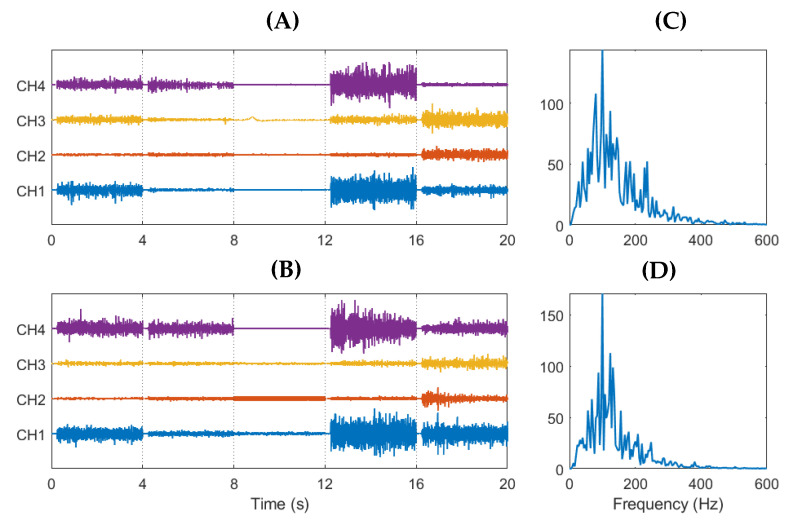
Example of recorded high-pass filtered signals from 4th gel and embroidered offline sessions of subject 1. EMG from gel electrodes (**A**) and embroidered electrodes (**B**). Power spectra of gel (**C**) and embroidered (**D**) signals. Samples of each gesture are four seconds in length, and gestures used, in order, are open hand, closed hand, relax, wrist extension, and wrist flexion. *Y*-axis are of arbitrary units.

The results from the usability test, found in Table 3, show similar performance between electrode types, with a throughput of 0.30 bit/s for both gel and embroidered electrodes. There is a slight variation in throughput across individual subjects for both electrode types. Completion rates are slightly higher for the textile electrodes across the three subjects, with an average of 97.2 ± 3.0% for the embroidered electrodes and 95.4 ± 5.7% for the gel electrodes. Examples of high-pass filtered EMG recordings and power spectra from both types of electrodes are shown in Figure 6. The spectra reveal similar frequency characteristics in both the textile and gel electrodes. Both have peak frequencies around 100 Hz and trail to zero above 500 Hz (note that no 50 Hz power line noise filtering is applied to the signals).

## 4. Discussion

This study compares the performance of embroidered textile EMG electrodes with disposable gel EMG electrodes in gesture recognition and real-time usability tasks with a view towards use in affordable upper limb prostheses. The results demonstrate that embroidered electrodes can achieve comparable performance to gel electrodes both online and offline. The general classification models of nine hand and wrist gestures trained on data from all three subjects achieved an average accuracy of approximately 96% for the embroidered and 97% for the gel electrodes. In contrast, the individual subject-specific models achieved an average of 98% accuracy for both electrode types, although the accuracy for the embroidered electrodes is slightly lower in both cases. The lower accuracy for the models trained on all data was expected as they are more generalised than the individual models. The accuracy would be expected to improve with a larger sample of subjects, as the models would become less dependent on subject variation and noise. The individual models trained using embroidered electrodes also showed slightly higher variability than the gel electrodes (Table 2), which may be due to a lower signal-to-noise ratio (SNR) in the embroidered type. The usability of the embroidered and gel electrodes was also shown to be similar as measured by throughput and completion rate in the Fitts’ law-based reaching task. The difference in completion rate between electrode types may be a consequence of the limited number of participants.

Although the results of this investigation are promising, future work is required to determine the suitability of embroidered electrodes to myoelectric control as there were several limitations to this pilot study. Examples of limitations include the small number of subjects, the use of subjects with intact limbs only, and lack of gender balance (two female subjects and one male), making it difficult to generalise the findings to real-world situations. Each subject was also only tested in a single session, so the study does not account for the variation of EMG signal over time due to natural biological fluctuations and the potential degradation of the fabric electrodes.

Another limiting factor was that the electrodes had to be secured in place using tape and tubing to ensure good skin contact and prevent motion artefacts, which made the applied pressure on the electrodes difficult to control and may have produced differences in signal quality between electrodes and subjects. This method of securing the electrodes also differs significantly from how the electrodes would be implemented in an actual prosthesis, making it difficult to generalise the results to practical myoelectric control. Pressure sensors combined with adjustable bands could have been used to ensure consistent pressure on the electrodes while maintaining good contact. Using such a setup, the sensitivity of the fabric electrodes to motion artefacts could be investigated, allowing an optimal level of pressure to be found that balances signal quality with user comfort.

The experiment is also limited by the electrode design because the 20 mm diameter of the electrodes restricted the number that could be applied to the forearm and made it difficult to secure them in place comfortably. Two-dimensional high-density EMG electrode arrays have shown promise for myoelectric control in recent years, including superior positional shift robustness and classification accuracy [21]. Such an arrangement has been proven viable with textile electrodes [11]. Implementing the embroidered electrodes in a high-density array form may therefore have improved performance.

Future work could also be conducted to analyse the potential for practical use in commercial myoelectric control. Although the classification algorithm used in the experiment is typical of those used in current sequential commercial prosthetics, testing with a simultaneous and proportional control algorithm would have better-assessed serviceability in future prosthetics. The assessment of applicability in myoelectric control could also have been improved by testing on amputee subjects rather than able-bodied subjects and by using an active prosthetic in an actual real-time reaching test rather than using a reaching simulation. It could also be improved by testing with the electrodes embedded into a fabric socket, garment, or sleeve, similar to how it would need to be implemented in a commercial device. This would allow many of the limiting factors to be investigated simultaneously, including the ease of placement for users, the susceptibility to motion artefacts, and user comfort.

Another necessary area of investigation would be analysis of the durability of the fabric electrodes during long-term use, such as would be expected of electrodes in prosthetics, and should include measuring the evolution of skin-electrode impedance and SNR over time. The effects of environmental factors, such as temperature and moisture, on signal quality also require investigation.

Despite these limitations, the results are in line with previous studies showing that using embroidered electrodes can achieve similar performance with gel sensors in upper limb movement classification for control of myoelectric prostheses [11,16,21,22]. Besides EMG, the embroidered electrodes also show promise for use in electroencephalography (EEG) [23] and electrocardiography (ECG) [24,25]. For instance, a recent study using the same type of embroidered electrodes for ECG measurement [26] showed that embroidered electrodes can capture high quality ECG signals, albeit with less stability than gel electrodes due to issues with skin contact. Some studies, such as [27], have investigated the possibility of using textile EMG electrodes in elasticated fabric bands to ensure skin contact and improve wearability in prosthetics, which is another potential development for embroidered electrodes.

## Figures and Tables

**Table 1:** Target configurations with associated difficulties and targets numbers.

Target Configuration	Distance from Origin	Target Width	Index of Difficulty	Associated Target Number
1	50	5	3.46	1, 7, 13, 19
2	50	10	2.59	2, 8, 14, 20
3	50	20	1.81	3, 9, 15, 21
4	100	5	4.39	4, 10, 16, 22
5	100	10	3.46	5, 11, 17, 23
6	100	20	2.59	6, 12, 18, 24

**Table 2:** Percentage classification accuracies of models trained on individual subject data for the recognition of nine gestures. Results are mean ± standard deviation over ten folds.

Subject	Electrode Type	
	Gel	Fabric
1	99.55 ± 0.29	99.23 ± 0.36
2	99.51 ± 0.58	95.63 ± 1.61
3	96.05 ± 1.06	98.74 ± 0.58
Mean	98.37 ± 2.01	97.87 ± 1.95

**Table 3:** The mean and standard deviation of throughput (TP) for each experimental subject and overall during the usability test.

Subject	Electrode Type	
	Gel	Fabric
1	0.31 ± 0.08	0.29 ± 0.08
2	0.29 ± 0.09	0.30 ± 0.09
3	0.29 ± 0.08	0.30 ± 0.09
All	0.30 ± 0.01	0.30 ± 0.01

## Data Availability

Data sharing is available per request.
